# Elimination of Interface Energy Barriers Using Dendrimer Polyelectrolytes with Fractal Geometry

**DOI:** 10.1021/acsami.3c01930

**Published:** 2023-06-03

**Authors:** E. Ros, T. Tom, P. Ortega, I. Martin, E. Maggi, J. M. Asensi, J. López-Vidrier, E. Saucedo, J. Bertomeu, J. Puigdollers, C. Voz

**Affiliations:** † Departament d’Enginyeria Electrònica, Universitat Politècnica de Catalunya (UPC), Barcelona 08034, Spain; ‡ Departament de Física Aplicada, Universitat de Barcelona, Martí i Franquès 1, 08028 Barcelona, Spain; § Institute of Nanoscience and Nanotechnology (IN2UB), Universitat de Barcelona, Barcelona 08028, Spain

**Keywords:** dipole, dipole film, conjugated polyelectrolytes, dendrimer, solar cells, Fermi-level pinning, electronic transport

## Abstract

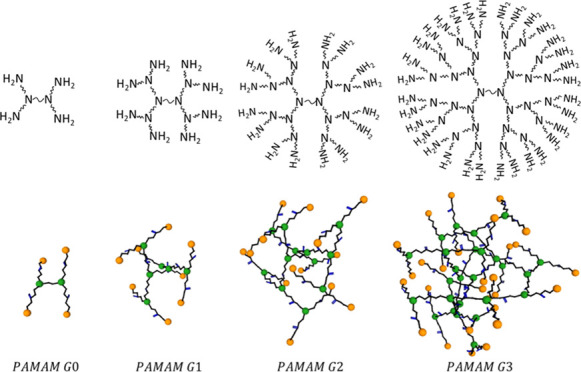

In this work we study conjugated polyelectrolyte (CPE) films based on polyamidoamine (PAMAM) dendrimers of generations G1 and G3. These fractal macromolecules are compared to branched polyethylenimine (b-PEI) polymer using methanol as the solvent. All of these materials present a high density of amino groups, which protonated by methoxide counter-anions create strong dipolar interfaces. The vacuum level shift associated to these films on n-type silicon was 0.93 eV for b-PEI, 0.72 eV for PAMAM G1 and 1.07 eV for PAMAM G3. These surface potentials were enough to overcome Fermi level pinning, which is a typical limitation of aluminium contacts on n-type silicon. A specific contact resistance as low as 20 mΩ·cm^2^ was achieved with PAMAM G3, in agreement with the higher surface potential of this material. Good electron transport properties were also obtained for the other materials. Proof-of-concept silicon solar cells combining vanadium oxide as a hole-selective contact with these new electron transport layers have been fabricated and compared. The solar cell with PAMAM G3 surpassed 15% conversion efficiency with an overall increase of all the photovoltaic parameters. The performance of these devices correlates with compositional and nanostructural studies of the different CPE films. Particularly, a figure-of-merit (*V*_σ_) for CPE films that considers the number of protonated amino groups per macromolecule has been introduced. The fractal geometry of dendrimers leads to a geometric increase in the number of amino groups per generation. Thus, investigation of dendrimer macromolecules seems a very good strategy to design CPE films with enhanced charge-carrier selectivity.

## 1 Introduction

**Figure 1:**
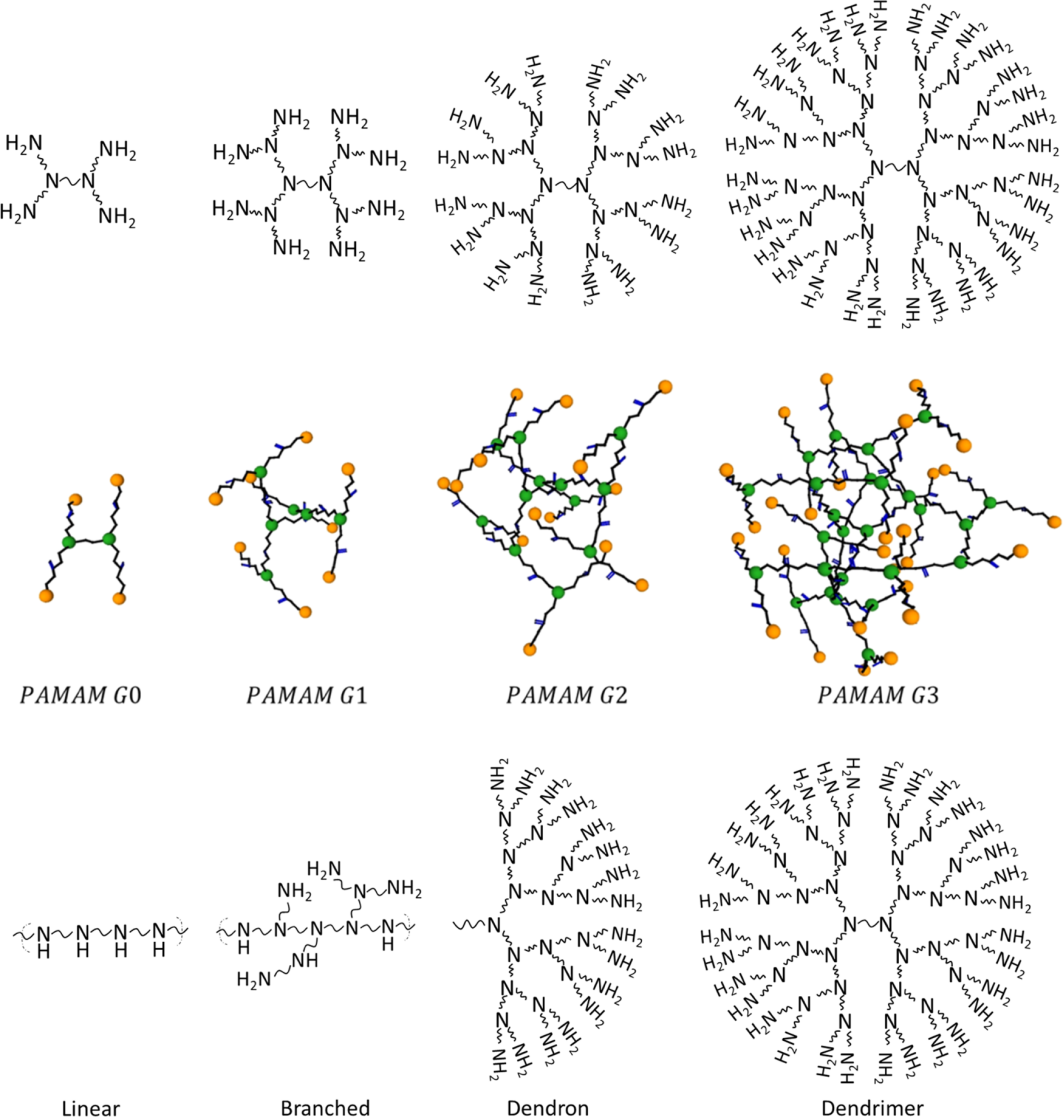
Schematic representation of PAMAM dendrimers for consecutive generations (top), Schematic view of the different geometry from linear polymers to branched, dendrons and finally dendrimers (bottom).

In the last years, a particularly active research field has been focused on developing alternative selective contacts for electronic devices. A clear example is the effort devoted to the development of dopant-free crystalline silicon (c-Si) solar cells. This interest is boosted by performance-limiting energy losses of conventional device structures. Heavily-doped diffused junctions increase recombination and may cause optical losses.^1−4^ Parasitical optical absorption at the front junction is also a concern in Silicon heterojunction (SHJ) solar cells.^5^ The use of hazardous gas precursors increases complexity and production cost.^6^ These issues have triggered the study of alternative carrier-selective layers for high-efficiency photovoltaic (PV) devices. These layers must be very transparent, besides providing good surface passivation, carrier selectivity and low contact resistance. All these effects should be ideally achieved by a cost-effective deposition method. Materials such as metal oxides,^7−10^ metal nitrides,^11−13^ alkali and alkaline-earth metal salts^14−16^ and organic polymers^17,18^ have been employed so far as selective contacts with remarkable results. In this context, there have been some reports on the use of dendrimers in photovoltaic devices.^19−21^ Dendrimers are highly ordered branched polymers, also referred as arborols or cascade molecules in the literature.^22−24^ The structure of dendrimers is typically tree-like with molecular units emanating from a core monomer.^25,26^ A branched replication of these molecular units leads to successive dendrimer generations (Figure 1). Each new generation surface groups becomes the branching points for new ramifications of the tree like molecule. This is performed by the addition to the molecule of a repeating unit that branches out. From the core can emerge a different number of branches that will define the shape of the macromolecule. Some unique properties of dendrimers such as solubility, nano-scaled size and low viscosity, allow a simple solution-process in a large variety of applications.^27−29^ Particularly, dendrimers have been used with good results in emerging PV technologies such as organic,^30−32^ dye-sensitized^33−35^ and perovskite solar cells.^36−38^ Nevertheless, valuable implementations in silicon heterojunction solar cells are still to be explored. In this sense, this work investigates a possible application of polyamidoamine (PAMAM) dendrimers in silicon heterojunction devices.^26^ Particularly, two PAMAM generations (G1 and G3) are studied to fabricate electron-selective contacts based on these dendrimers. The quality of these contacts is compared to that achieved using branched polyethylenimine (b-PEI) solutions. This other polymer has been extensively reported as a cathode interlayer to improve electron extraction for different PV technologies that include silicon solar cells.^18,39−41^ As in the case of PEI and similar cationic polymers,^42,43^ the proposed working principle for PAMAM assumes the formation of a conjugated polyelectrolyte with the solvent.^44,45^ Then, self-assembled monolayers with a strong dipolar moment are formed at the interface with the metallic electrode. Consequently, electron transport benefits from an effectively reduced metal work function. The working principle of these electron-selective contacts will be described in detail throughout this article.

## 2 Experimental Section

PAMAM dendrimers with ethylenediamine core, amide branches and amine functionality were studied for interface modification of aluminum (Al) contacts on n-type c-Si. This electrode can be used as a passivated electron selective contact for c-Si solar cells. Dendrimer solutions of different weight percentages between 0.001 and 0.1 wt % were prepared using methanol as the solvent. All the chemicals were purchased from Sigma- Aldrich.^46^ Dendrimer solutions of two generations (G1 and G3) were spin-coated at 5000 rpm for 30 s on silicon and sapphire substrates. Following, annealing on a hot plate for 30 s at 90 °C in ambient atmosphere dried the solvent to obtain nanometric films of the dendrimers. Similarly, samples of the b-PEI polymer were prepared as described in a previous work.^41^

The samples on silicon were prepared on polished FZ n-type (2 Ω·cm) wafers of thickness 280 μm and (100) crystalline orientation. All the wafers were treated in 1% diluted hydrofluoric acid (HF) for 1 min to obtain an oxide-free silicon surface before spin-coating the dendrimer solutions. The thickness of the films was varied by using solutions with different dendrimer concentration. After evaporating the solvent, the nanometric thickness of the films was measured by means of a homemade ellipsometer.^47^ Subsequently, the effect of interface modification on the specific contact resistance of Al/n-type c-Si electrodes was studied by the transfer length method (TLM). For these measurements, an Al layer 300 nm thick was evaporated using a shadow mask to define a set of contacts separated between 150 and 500 μm. Optical characterization of dendrimer films on sapphire glass substrates was done using an UV–visible-NIR Lambda 950 spectrophotometer (Perkin Elmer, Shelton, CT). Atomic force microscopy (AFM) images were obtained using a Multimode Nanoscope 8 system (Bruker, Santa Barbara, CA) to study the surface morphology of the films on silicon. XPS and UPS spectra were measured with a Phoibos 150 analyzer (SPECS GmbH, Berlin, Germany) in ultra-high vacuum conditions (base pressure <5 × 10^–10^ mbar). A monochromatic K-α (1486.74 eV) X-ray source and an UV He I lamp (21.2 eV) were used as excitation for XPS and UPS measurements, respectively. The resolution in the binding energy was estimated as the full-width-at-half-maximum (FWHM) of the 3d_5/2_ peak for a sputtered silver (Ag) layer, which was 0.62 eV for XPS and 0.11 eV for UPS spectra. The relevant nitrogen (N), carbon (C) and oxygen (O) peaks were deconvoluted using the CasaXPS software.^48^ The work function of the silicon surface coated by the dendrimer films was calculated from the secondary-electron cut-off of UPS spectra.^49^ The nanostructure of the films was studied by high-resolution transmission electron microscopy (HRTEM) using a FEI Titan system (60–300 kV).^50^ Besides, the chemical composition was analysed with subnanometer spatial resolution by electron energy loss spectroscopy (EELS).

Electron-selective contacts on silicon using b-PEI polymer and PAMAM (G1 and G3) dendrimers for interface modification of Al cathodes were studied. The structure of these solar cells is shown in the Supporting Information of this work. All these devices were fabricated on polished n-type silicon wafers. First, the dendrimer films were spin-coated on the rear side and dried during a short annealing step. Immediately, the dendrimer films were coated by a 300 nm thick Al contact evaporated in vacuum. Then, a thin vanadium oxide (V_2_O_5_) layer (4 nm) was grown on the front side by atomic layer deposition (ALD). A detailed study of this hole-selective contact as a doping-free alternative for silicon-based solar cells can be found in the literature.^9,10^ Subsequently, a 75 nm thick indium-tin-oxide (ITO) layer was deposited by RF magnetron sputtering as a front transparent electrode and antireflection coating. The active area of the solar cells (4 cm^2^) was defined following standard photolithographic and wet-etching steps. Finally, a front contact Ag grid of 2 μm was thermally evaporated using a shadow mask. The metallic grid covered 4% of the active area in the solar cells. The current density-voltage (*J*–*V*) electrical characteristics were measured under standard conditions (100 mW/cm^2^, AM 1.5G spectrum) using a 94041A solar simulator (Newport, Irvine, CA). Finally, the external quantum efficiency curves were obtained with a QEX10 set-up (PV Measurements, Point Roberts, WA).

Measurements performed on different devices were reported as average ± standard deviation (SD). In all cases, significance was defined as *p* ≤ 0.05. Statistical analysis was conducted using GraphPad Prism software.

## 3 Results and Discussion

**Figure 2:**
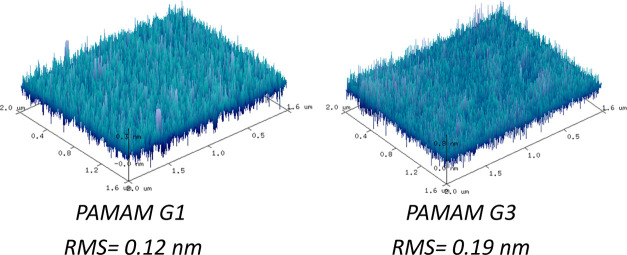
Atomic Force Microscopy topographic images of CPE films based on PAMAM G1 and G3 spin-coated on silicon substrates.

**Figure 3:**
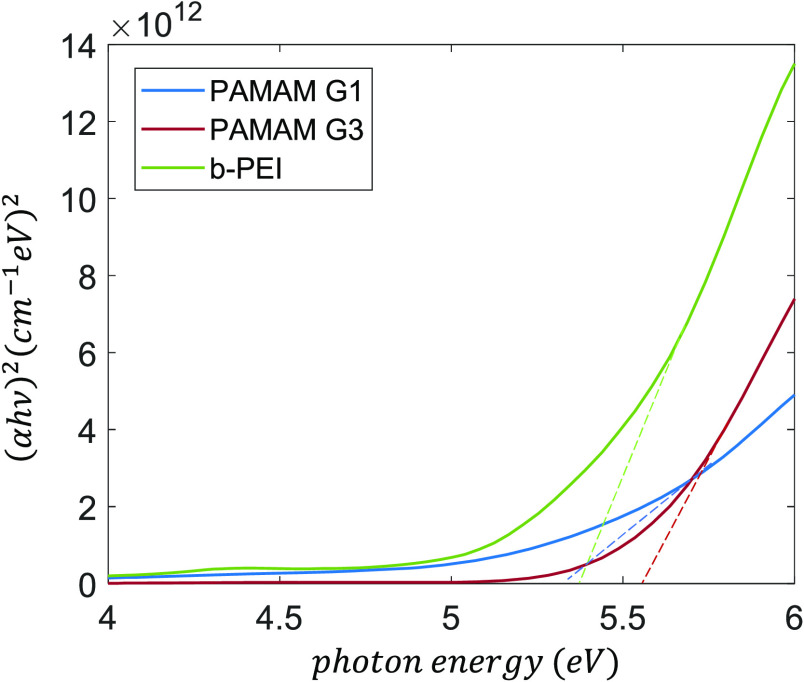
Tauc plot to calculate the bandgap of CPE films based on b-PEI, PAMAM G1 and G3. These curves are calculated from transmittance measurements of the films deposited on sapphire substrates.

In this work we focus on the characterization of PAMAM dendrimer films (generations G1 and G3). The polymer b-PEI that is used here as a reference was extensively studied in a previous work.^41^ AFM images (Figure 2) show that spin-coated PAMAM films are very smooth. The room mean square roughness (Sq) for G1 and G3 films were only 0.12 and 0.19 nm, as compared to the 0.3 nm of b-PEI films (Supporting Information). The reduced Sq values of the dendrimers could be attributed to their different nature, because the solvent, weight concentration, spinner velocity and post-anneal treatment were kept identical. All the films under study were less than 4 nm thick, even the ones obtained from more concentrated solutions. Moreover, they are practically non-absorbent in the visible range according to the large optical gaps (*E*_gap_) obtained from the Tauc plot (Figure 3). In the case of PAMAM dendrimers the *E*_gap_ values were 5.36 and 5.56 eV for G1 and G3, respectively. An intermediate *E*_gap_ value of 5.41 eV was measured for the polymer b-PEI. Such a high bandgap suggests an insulator behavior, which would explain the need of ultra-thin films favoring electron tunneling as it was previously reported.^41^

The idea of using PAMAM dendrimers to fabricate electron selective contacts arises from the large number of amino groups in their macromolecules. Solution processing of these materials leads to the formation of so-called conjugated polyelectrolytes (CPE).^43−45^ In our case, methanol behaves as a weak Brønsted acid that protonates the amino groups.^51^ This phenomenon has already been observed in other conjugated polyelectrolytes using solvents with higher dissociation constants than methanol, moreover it has been associated to the Lewis basicity of these high amino containing polymers.^61^ Then, electrostatic adhesion of methoxide counter anions to protonated amino groups results in dipolar CPE embodiments (R-NH_3_^+^:CH_3_O^–^). Self-assembling of the CPE film with certain orientation and dipole density finally modifies the work function at the interface. The strength of this effect can be primarily measured by Ultraviolet Photoelectron Spectroscopy (UPS).^52^

**Figure 4:**
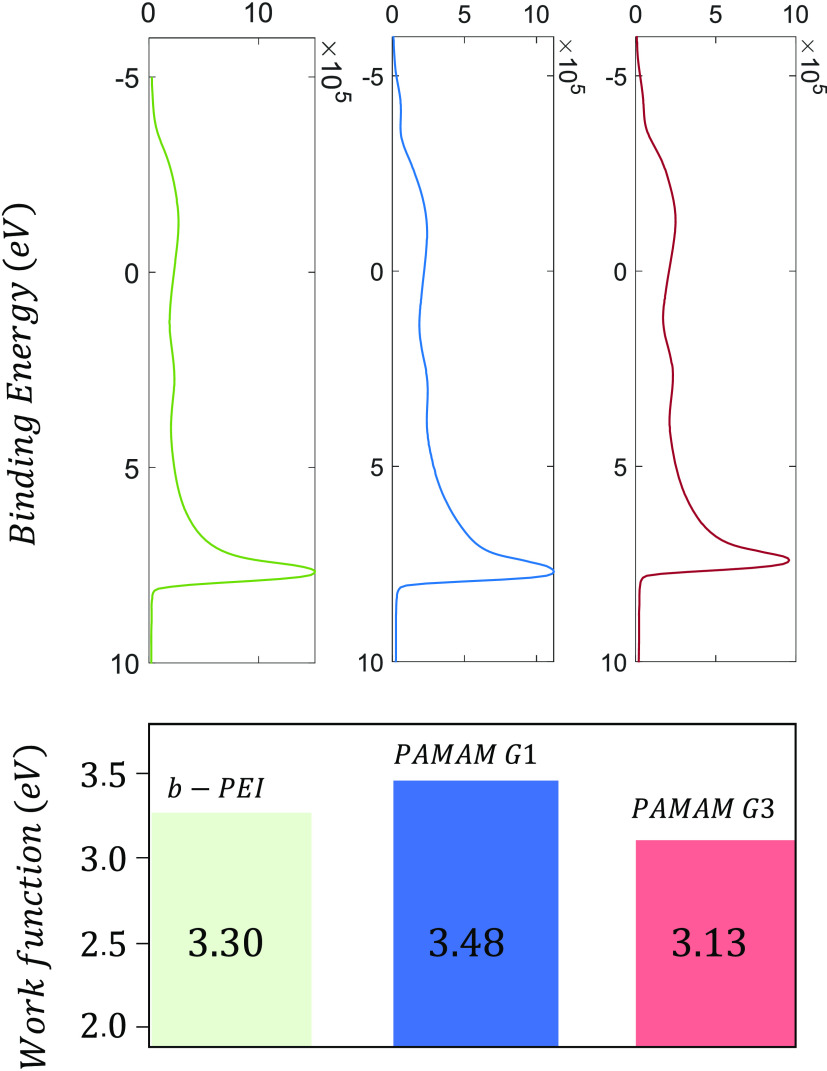
UPS spectra of n-type silicon substrates coated by CPE films of b-PEI, PAMAM G1 and G3 (up). Work function values of each sample calculated according to *WF* = *hν* – (*E*_cutoff_ – *E*_onset_) – *qV*_bias_ (eq 1).

Figure 4 shows the UPS spectra measured for n-type silicon substrates coated with films of b-PEI and both G1 and G3 PAMAM generations. The work function (WF) of these samples can be calculated as^49^
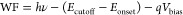
1where the photon energy *hν* is 21.2 eV (He I) and the width of the UPS spectra is given by the difference between the measured secondary-electron cut-off and onset energy levels. The bias voltage applied to the sample must be also considered, which in these measurements was *qV*_bias_ = 10 eV. In all cases the calculated WF values where significantly reduced with respect to the value around 4.2 eV expected for 2 Ω·cm n-type silicon. Considering that the films are a few nanometers thick, such changes in the work function can be attributed to a surface potential (Δ*V*). This effect is a consequence of the dipolar interface that results from self-assembling in the CPE film. The vacuum level shift (*q*Δ*V*) measured in the WF values were 0.93, 0.72, and 1.07 eV for b-PEI and G1 and G3 PAMAM generations, respectively. Therefore, the weaker modification corresponds to PAMAM G1 while the most intense effect is measured for G3. The surface potential for b-PEI lays between the values of the two dendrimer generations.

Dipolar interface layers effectively shift the electrode work function to higher/lower WF values that finally improve the operation of hole/electron selective contacts. Equilibrium in metal/semiconductor structures involves charge transfer until alignment of Fermi level across the junction. In particular, electrons are transferred from low work function metals to the semiconductor. The dipolar interlayer intercalated between the metal and the semiconductor also contributes to reduce metal-induced-gap-states (MIGS).^53−55^ As a consequence, suppression of Fermi level pinning brings the semiconductor surface into electron accumulation. This situation yields a low contact resistance with optimum electron injection/extraction at the corresponding electrode.^56,57^ The working principle can be summarized as follows. Dipolar films at the interface modify the work function of the electrode by introducing a surface potential (vacuum level shift). This effect depends on the magnitude, density and orientation of intermolecular dipoles formed in the CPE film. As a result, aluminium can perform as well as magnesium or calcium electrodes for electron-selective contacts. A similar approach with adequate CPE films could work to replace expensive high work function metals like platinum or gold in hole-selective contacts.

**Figure 5:**
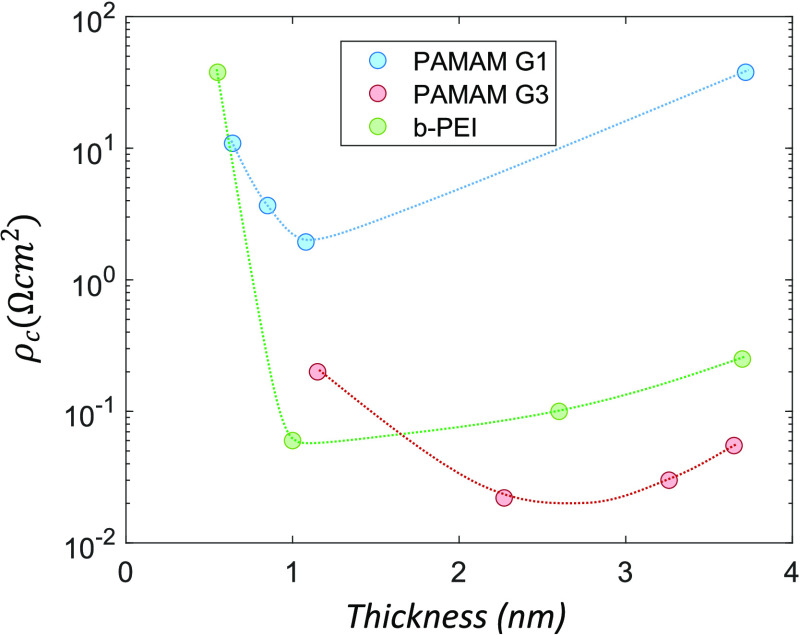
Specific contact resistance as a function of the film thickness for conjugated polyelectrolytes based on b-PEI, PAMAM G1 and G3. In all cases a minimum thickness is needed to surpass Fermi level pinning and reduce the contact resistance. The contact resistance increases for thicker layers, suggesting that the main transport mechanism is direct electron tunneling.

Figure 5 shows the specific contact resistance (ρ_c_) calculated from TLM electrical measurements as a function of the film thickness for b-PEI, G1 and G3 PAMAM generations. As it can be observed, the thickness of the dipolar CPE films is very important for the final electrical performance of the stack with the aluminium contact. A minimum thickness is needed for depinning the Fermi level, as deduced from the sharp increase in contact resistance of the thinnest samples. Sub-nanometric films do not sustain the required surface potential or they cannot mitigate metal-induced gap states. Nevertheless, the minimum contact resistance is already achieved with very thin layers. Then, on the right-hand side of Figure 5 the contact resistance increases again for thicker films. This behaviour suggests direct tunneling as the dominant conduction mechanism, as it has been reported for b-PEI in a previous work.^41^ PAMAM G3 yields the lowest values of contact resistance and it is less influenced by the layer thickness. There is also good correlation between the surface potentials measured by UPS and the optimum (minimum) contact resistance values of each material. The higher WF reduction of PAMAM G3 (1.07 eV) leads to the lowest ρ_c_ value of 20 mΩ·cm^2^, followed by b-PEI (0.93 eV) that gives 60 mΩ·cm^2^. Finally, the weakest effect of G1 (0.72 eV) increases the contact resistance to a significantly higher value of 1.9 Ω·cm^2^.

The suitability of different conjugated polyelectrolytes to be used in selective contacts relies on the factors that may affect their surface potential. For instance, all the organic materials studied in this work share the presence of amino groups (aminos) in their molecular structure. The polymer b-PEI has 4 amino groups per monomer, whereas PAMAM G1 has 8 amino groups that increase to 32 for PAMAM G3. However, TLM measurements show that b-PEI performs better than PAMAM G1 and its surface potential was also higher. Evidently, the absolute number of amino groups per molecular unit is not the only factor determining the surface potential. First, these amino groups must be protonated to form dipoles by counter-anion condensation. Then, the density and orientation of these dipoles is also very relevant in the CPE film.

**Figure 6:**
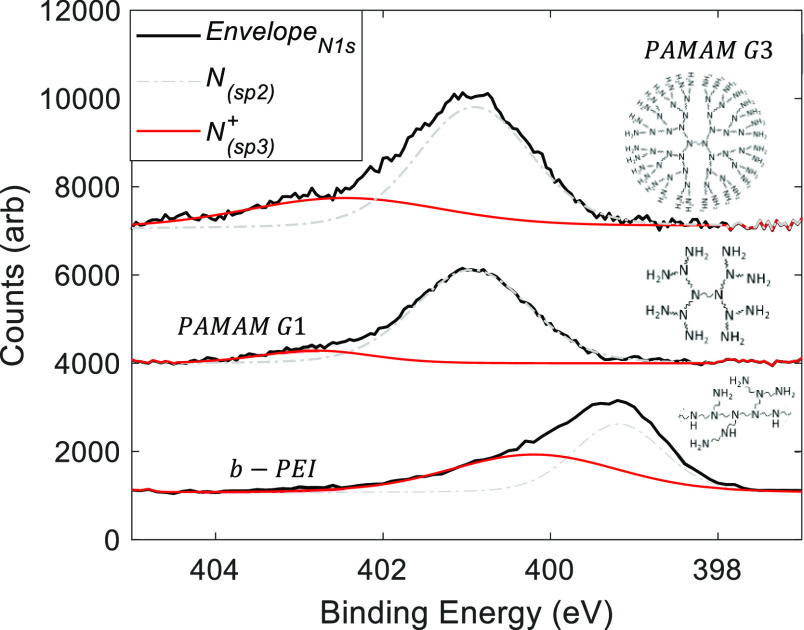
Deconvolution of nitrogen signal in XPS spectra measured on CPE films based on b-PEI, PAMAM G1 and G3. This analysis evaluates the fraction of amino groups protonated in these films.

Compositional analysis by XPS targeted the different hybridization forms of nitrogen in the films (see Figure 6). The spectrum of nitrogen can be deconvoluted into two Gaussians that fit well the envelope curve. The Gaussian centered at a higher binding energy corresponds to nitrogen hybridized as sp^3^ (quaternized), i.e., nitrogen in protonated amino groups.^58^ Hence, the ratio of protonated amino groups can be calculated as the area under this Gaussian (red line) divided by the total area under the curve. For instance, nitrogen in protonated amino groups is around 40% of the total nitrogen signal for b-PEI. The total count of nitrogen atoms per b-PEI monomer is 10, from which 4 are in surface amino groups. Consequently, one can say that all amino groups of b-PEI are effectively protonated. In PAMAM G1, protonated amino groups only contribute a 5% to the nitrogen signal. This percentage results in 1.3 average protonated amino groups, which is much less than the 8 amino groups contained in each G1 molecule. A similar calculation for PAMAM G3 (26% signal under red curve) gives 31.7 protonated amino groups, which is very close to the 32 amino groups present in this dendrimer. The calculation above explains why the contact resistance of PAMAM G1 is worse (higher) compared to that of b-PEI. Considering that only protonated amino groups form dipoles in the CPE film, b-PEI polymer is indeed a very good choice. Nevertheless, PAMAM G3 performed even better because of the higher number of surface amino groups. In this sense, the fractal geometry of dendrimers can be an adequate molecular design to achieve more intense dipolar interlayers.

**Figure 7:**
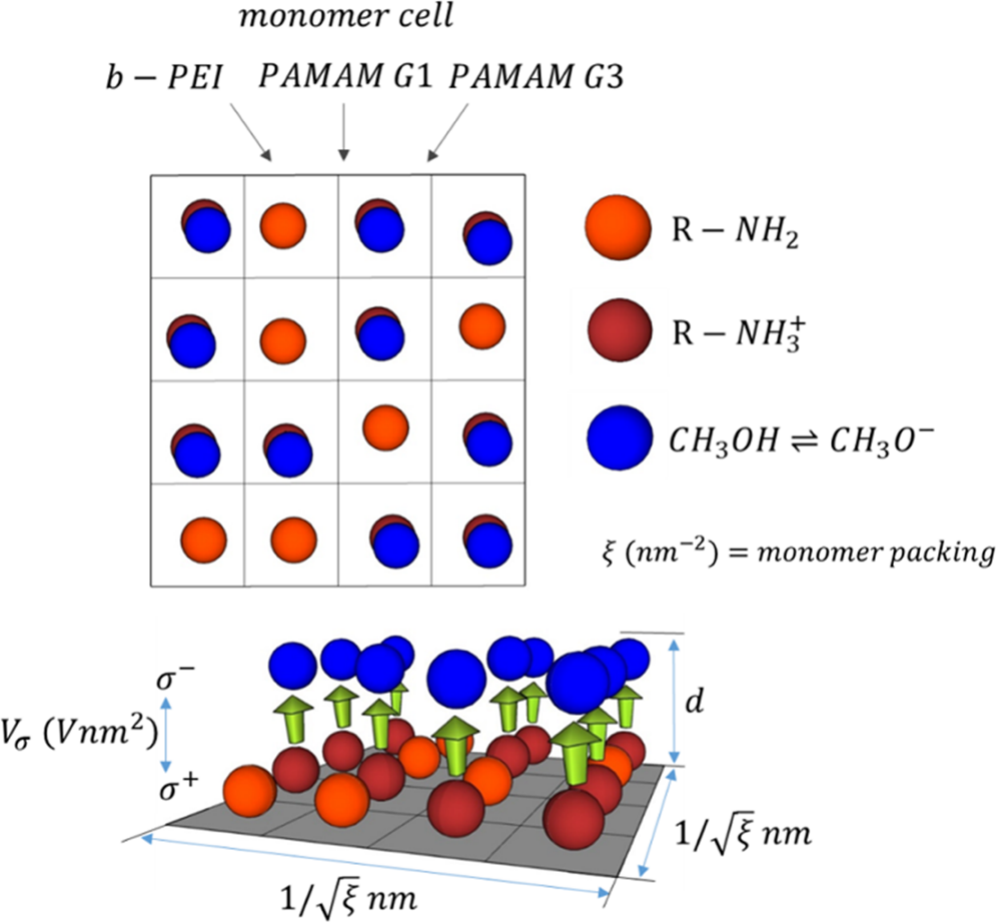
Schematic to explain the figure-of-merit *V*_σ_ and its relation to surface potential introduced by conjugated polyelectrolyte films.

The surface potential due to a dipolar layer can be calculated using the Helmholtz equation^59^
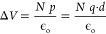
2where *N* is the number of dipoles per unit area (areal number density), *p* their net dipolar moment normal to the substrate and ϵ_o_ the dielectric constant of vacuum. Here, it is assumed that the surface potential is mainly due to oriented dipoles from the conjugated polyelectrolyte. The value of *N* can be expressed as the number of monomers/dendrimers per unit area (ξ) multiplied by the number of protonated amino groups (*n*^+^ value in Table 1). The packing factor ξ can be varied by adjusting the concentration and spin-coating conditions of the CPE film. Thus, it is convenient to introduce a figure-of-merit that we will call sigma-potential (*V*_σ_). This figure-of-merit can be defined as:
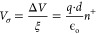
3which is intrinsic to the material and quantifies its ability to create strong dipolar layers. The surface potential can be calculated as the product of the packing factor of molecules and their corresponding figure-of-merit (Δ*V* = ξ·*V*_σ_). Then, *V*_σ_ turns out to be the surface potential per unit area due to each monomer/dendrimer. In eq 3 the distance between the charges of the dipole (*d*) can be considered the thickness of the film (see Table 1). Even if it were not a monolayer, inner dipoles would cancel out as for polarized dielectrics. Therefore, the conjugated polyelectrolyte results in positive and negative surface charge densities that spread between the semiconductor and metal electrode (Figure 7).

**Table 1:** Organic Materials Studied in This Work to Obtain Dipolar Layers^a^

material	total number of nitrogen atoms	*n*^+^, number of protonated aminos	total number of aminos	thickness (nm)	figure-of-merit *V*_σ_ (V·nm^2^)
PAMAM G1	26	1.3	8	1.1	26
b-PEI	10	4	4	1	72
PAMAM G3	122	31.7	32	2.3	1300

a Starting from the left column: total number of nitrogen atoms per monomer/dendrimer, average number of protonated amino groups per monomer/dendrimer (*n*^+^) calculated from XPS analysis, total number of amino groups per monomer/dendrimer, thickness of the optimized CPE film and, finally, figure-of-merit for the suitability of the material to make CPE films. In the column header amino groups has been shortened as aminos.

**Figure 8:**
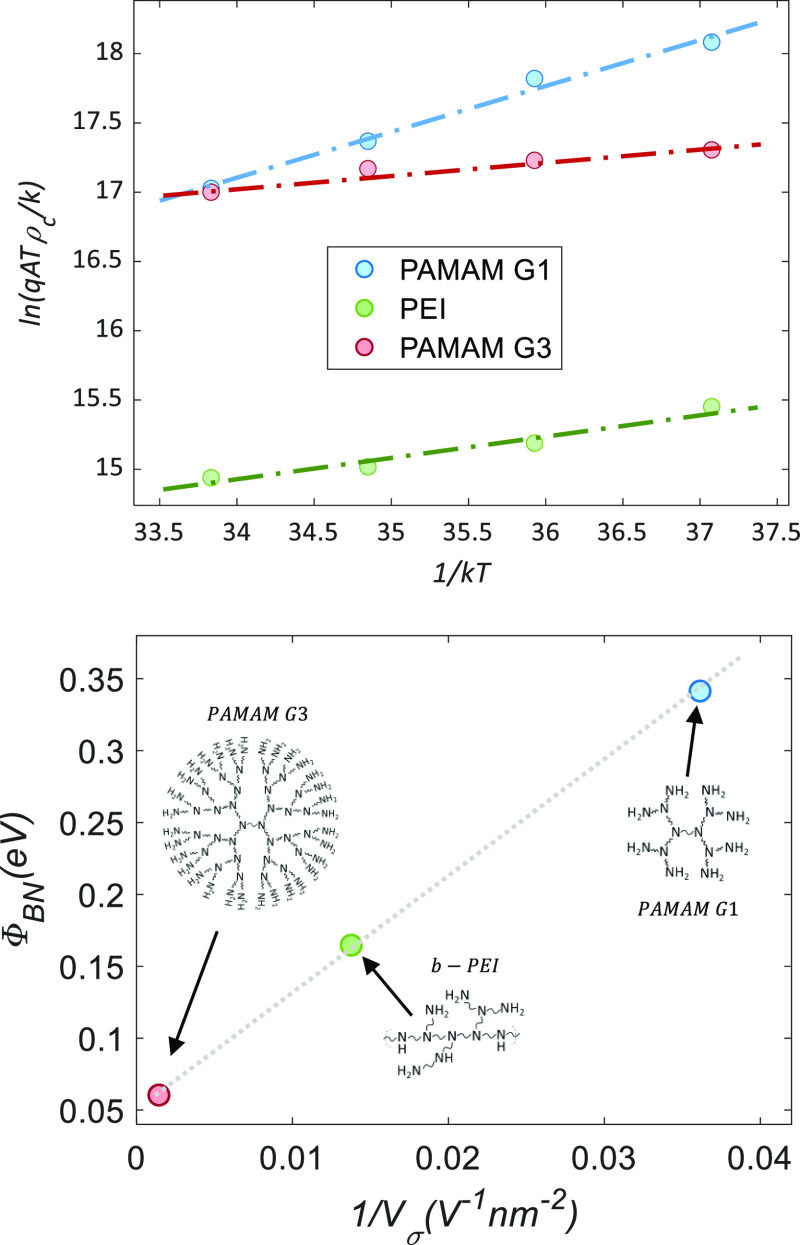
Thermal dependence of the contact resistance measured on the thinner CPE films of b-PEI, PAMAM G1 and G3 (top). Energy barrier of each same deduced from its thermal dependence (eq 4) plotted versus reciprocal *V*_σ_ (bottom).

The value of *V*_σ_ and hence the surface potential are proportional to the film thickness (eq 3). This dependence explains the increase of contact resistance for the samples with too thin CPE films. On the left-hand side of Figure 5, it can be assumed that thermionic emission over an energy barrier (ϕ_*B*_) is the mechanism limiting electronic transport. The contact resistance for such Schottky-like junction is given by the following equation
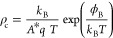
4where *k*_B_ is the Boltzmann constant, *q* the elementary charge, *T* the temperature in Kelvin and A* the Richardson constant.^60^ The energy barrier can be evaluated by doing TLM measurements at different temperatures. The ρ_c_ values extracted from these measurements (Figure 8, up) show a thermally activated behaviour, which can be fitted to calculate the energy barrier ϕ_B_ using eq 4. The high *V*_σ_ value of PAMAM G3 (Table 1) traduces into a negligible energy barrier of about 0.05 eV. PAMAM G1 with a much lower *V*_σ_ value results in a high energy barrier close to 0.35 eV. Again, b-PEI polymer falls midway the two dendrimer generations. It is appropriate to point out that these ϕ_B_ values are measured on excessively thin films (left-hand side of Figure 5). The energy barrier of films with optimized thickness (see Table 1) would be lower, as it is deduced from the measured contact resistances. Anyhow, these measurements confirm that the sigma-potential *V*_σ_ is indeed a good figure-of-merit. The energy barrier ϕ_B_ is inversely proportional to *V*_σ_ with quite good approximation (Figure 8, down). It could be argued that *V*_σ_ only weights the number of protonated amino groups per monomer/dendrimer and the effect of the film thickness. Note that the surface potential depends also on the number of monomer/dendrimer molecules per unit area, i.e., the value of the packing factor ξ in eq 3. Nevertheless, the clear trend observed in Figure 8 (down) suggests that this factor does not make significant differences between b-PEI, PAMAM G1 and G3. This could be a consequence of the partially random location of dipoles achieved by spincoating deposition technique resulting in a dipole arrangement similar to a spin glass. However, the situation could change for conjugated polyelectrolites based on other materials or using different solvents.

**Figure 9:**
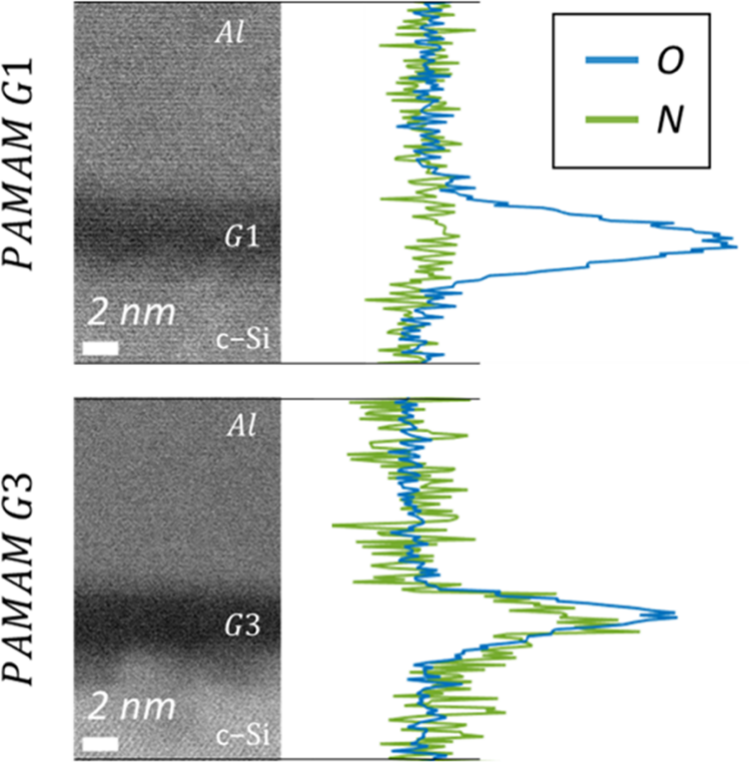
HRTEM cross-section images comparing CPE films of PAMAM G1 and G3 dendrimers. On the right side, EELS compositional analysis by EELS to detect nitrogen and oxygen content in the films.

Apart from evaluating b-PEI as a good polymer reference, this work specifically compares two PAMAM generations. The fractal geometry of these dendrimers can be used to increase the number amino groups, which seems a good strategy to obtain stronger dipolar layers. Then, films of both PAMAM generations have been studied by analytical HRTEM. Figure 9 compares cross-section images of G1 and G3 films both about 2 nm thick. Electron Diffraction Spectroscopy (EDS) measurements did not detect the low amount of nitrogen present in any of these layers. However, electron energy loss spectroscopy (EELS) was able to resolve oxygen and nitrogen signals with subnanometric spatial resolution. The peak of oxygen extends over the dark region that corresponds to PAMAM films between silicon and the metallic contact. This signal could have some contribution of oxidation effects or solvent residues, but is mainly attributed to methoxide counterions in the CPE film. Nitrogen was hardly detectable in PAMAM G1, but a very clear peak was resolved for G3. Both oxygen and nitrogen atoms are involved in the dipoles (R-NH_3_^+^:CH_3_O^–^) used to explain the operation of CPE films. The distinct and slightly shifted peaks of PAMAM G3 agree with its higher surface potential, which finally leads to better electron transport. The stronger nitrogen signal of PAMAM G3 compared to G1 can be explained by the much higher content of this element in its macromolecule (Table 1). Moreover, signal detection in the very thin lamellas used for EELS analysis can be much easier in highly ordered CPE films. This seems to be the case of PAMAM G3 according to the different characterizations done on this work.

**Figure 10:**
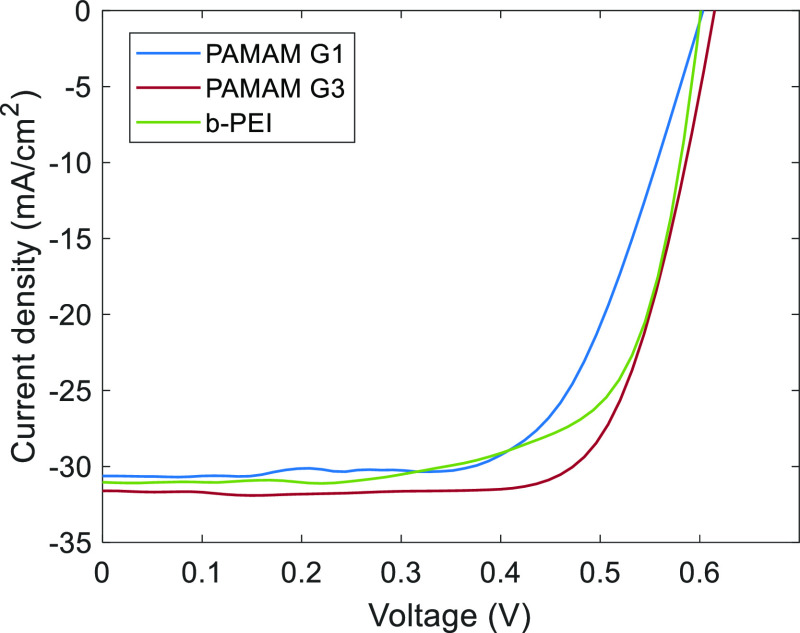
Electrical characteristics (JV curves) measured under AM1.5 illumination for the solar cells implementing electron-selective contacts based on CPE films of the materials studied in this work.

Finally, the different materials studied in this work were tested in proof-of-concept photovoltaic devices. Particularly, solar cells on n-type c-Si were fabricated using rear side electron-selective contacts based on these CPE films with an Al electrode. The thickness of the CPE films were those optimized to get minimum contact resistance values (Figure 5). The front side implemented a V_2_O_5_ hole-selective layer coated by an antireflection ITO transparent electrode with a final grid of Ag as the metallic contact. The structure of these solar cells has been described with some more detail in Section 2. Additional information on the use of V_2_O_5_ hole-selective contacts in different solar cell architectures can be found elsewhere.^9,10^ The electrical characteristics measured under reference AM1.5 illumination are shown in Figure 10. The corresponding photovoltaic parameters of these solar cells are summarized in. There is good correlation between the performance of the solar cells and the different results discussed along this manuscript.

The solar cell with PAMAM G3 was the best, surpassing 15% conversion efficiency. This result agrees with the higher surface potential and lowest contact resistance of this dendrimer. As for the rest of the experiments, PAMAM G1 gave the worst result (12.5%) with b-PEI performing in the middle (13.3%) of both dendrimer generations. Photovoltaic parameters are summarized in Table 2.

**Table 2:** Photovoltaic parameters of the proof-of-concept solar cells comparing the quality of the different materials to be used as electron-selective contacts in CPE films

material	*V*_oc_ (mV)	*J*_sc_ (mA/cm^2^)	*FF* (%)	*PCE* (%)
PAMAM G1	603 ± 1	30.8 ± 0.7	67.1 ± 0.9	12.5 ± 0.1
b-PEI	605 ± 3	31.1 ± 0.5	70.5 ± 0.5	13.3 ± 0.2
PAMAM G3	618 ± 2	32.7 ± 0.6	74.5 ± 0.3	15.1 ± 0.3

**Figure 11:**
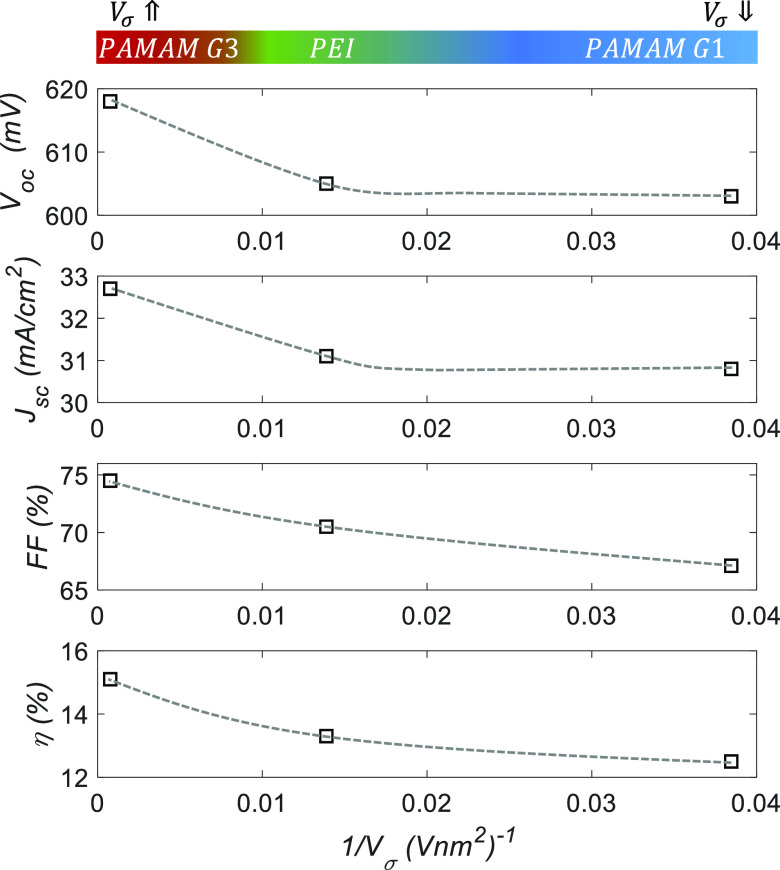
Photovoltaic parameters versus the figure-of-merit *V*_σ_ for the proof-of-concept solar cells fabricated in this work. These results confirm that *V*_σ_ is representative for the quality of the different CPE films to be used as electron-selective contacts.

Figure 11 plots all parameters of the measured *J*–*V* curves versus the figure-of-merit *V*_σ_ defined in this work (eq 3). The increase in efficiency is explained by a general improvement of all the PV values. It is particularly clear the effect on *FF*, which is typically limited by the parasitic series resistance. It has been already shown that the higher *V*_σ_, the lower is the contact resistance. A higher surface potential also leads to better surface passivation and charge-carrier selectivity. This happens because charge transfer from Al contact to the semiconductor brings the silicon surface to electron accumulation. Consequently, the *J*_sc_ and *V*_oc_ values also improved with *V*_σ_ when comparing the different materials.

## 4 Conclusions

Conjugated polyelectrolytes have been widely used to obtain selective contacts in new generation electronic devices. The operating principle is based on the formation of strong dipolar interlayers, which introduce a relatively high surface potential. As a consequence, modification of the work function at the electrode contributes to create charge-carrier selective contacts. In this work, the b-PEI polymer has been compared with two generations G1 and G3 of PAMAM dendrimer. In either of these cases, dipoles are formed by protonation of amino groups with electrostatic adhesion of metoxide counteranions from the solvent, which in our case was methanol. Electron-selective contacts based on these conjugated polyelectrolytes have been tested on n-type silicon with an aluminum contact. In all cases, a minimum thickness is required to eliminate Fermi level pinning. Then, charge transfer from the metal to the semiconductor takes the silicon surface to electron accumulation. This effect not only leads to excellent electron contacts, it also promotes charge-carrier selectivity (hole-blocking) that is good for many semiconductor devices. The contact resistance increases beyond an optimum thickness, suggesting that direct tunneling is the main electron transport mechanism. The Helmholtz equation to calculate the surface potential has been used to define a figure-of-merit *V*_σ_ for this family of conjugated polyelectrolytes. The sigma-potential takes into account the thickness of the film and, more importantly, the number of protonated amino groups per macromolecule. The fractal structure of dendrimers like PAMAM results in a geometric increase in the number of amino groups per macromolecule. In this sense, the use of dendrimers in conjugated polyelectrolytes seems an original strategy to obtain better selective-contacts. The possibilities are huge, PAMAM G1 has 8 surface amino groups, they increase to 32 in G3, 64 for G4, etc. Definitely it may be not such straightforward, because self-assembling in conjugated polyelectrolytes is equally essential. Besides a high density of amino groups, they must be protonated by the solvent to generate the dipole effect. Finally, good orientation of these dipoles at the interface is needed to create the surface potential that explains charge-carrier selectivity. Conceptually, all the methodology developed in this work could be applied to different dendrimers or for other functionalization groups. This could also apply to different electronic devices requiring enhanced electron transport through the interface between two materials.

## Supporting Information Available

In supporting information one can find complementary data and graphs relevant to the research. These include AFM measurements of PEI thin films. Measured energy barrier as a function of amino surface groups, as well as protonated amino groups per monomer unit corresponding to a hyperbolic dependence. Other graphs such as dependence of UPS workfunction shift with figure of merit sigma potential. Finally reference solar cell IV curve and PV parameters without any dipole at the interface between the semiconductor and the metal. And an schematic of the photovoltaic device test structure (PDF)
