# Supplementary material for: Elimination of Interface Energy Barriers Using Dendrimer Polyelectrolytes with Fractal Geometry
Source: ACS Appl Mater Interfaces. 2023 Jun 3;15(23):28705–15. doi: 10.1021/acsami.3c01930 (PMC10802975; doi:10.1021/acsami.3c01930)
Supplement: Supplementary file 1 — am3c01930_si_001.pdf [file am3c01930_si_001.pdf]

Supporting information

Elimination of interface energy barriers using dendrimer polyelectrolytes with fractal geometry

[1]E. Ros[*#], [2,3]T. Tom[*], [1]P. Rafael Ortega, [1]I. Martin, [1]E. Maggi, [2,3]J.M. Asensi, [2,3]J. López-Vidrier, [1]E. Saucedo, [2,3]J. Bertomeu, [1]J. Puigdollers, [1]C. Voz

[1] Departament d’Enginyeria Electrònica, Universitat Politècnica de Catalunya (UPC), Barcelona 08034, Spain

[2] Departament de Física Aplicada, Universitat de Barcelona, Martí i Franquès 1, 08028, Barcelona, Spain

[3] Institute of Nanoscience and Nanotechnology (IN2UB), Universitat de Barcelona, Barcelona, Spain

[*] Shared co-first authorship

[#] Corresponding autor: eloi.ros@upc.edu

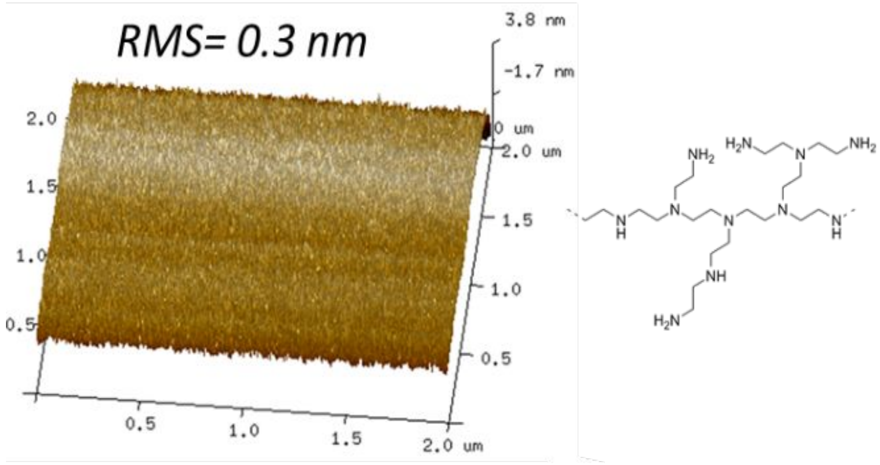

S1- AFM signal of a 4 square micron area for a Si/PEI structure.

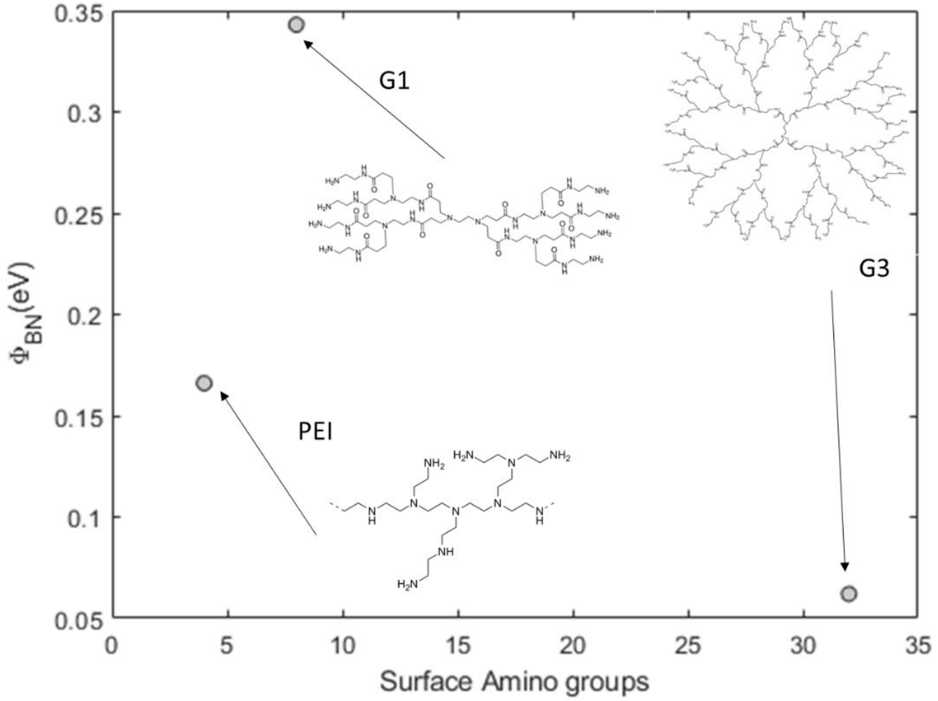

S2- Energy barriers as a function of each monomeric unit number of surface amino group.

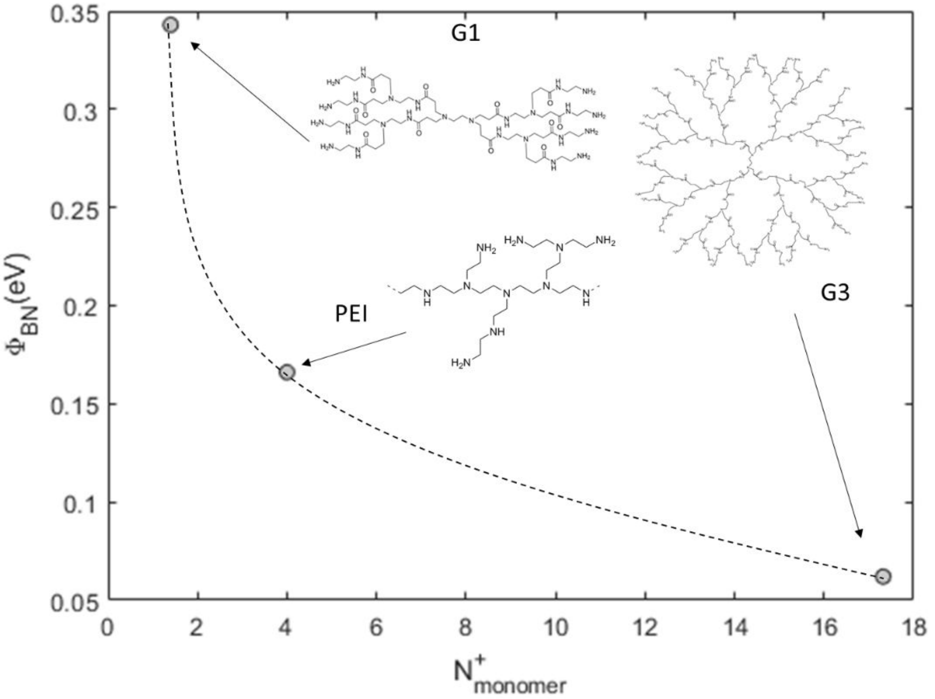

S3- Energy barrier as a function of the protonated amino surface groups in each polymer

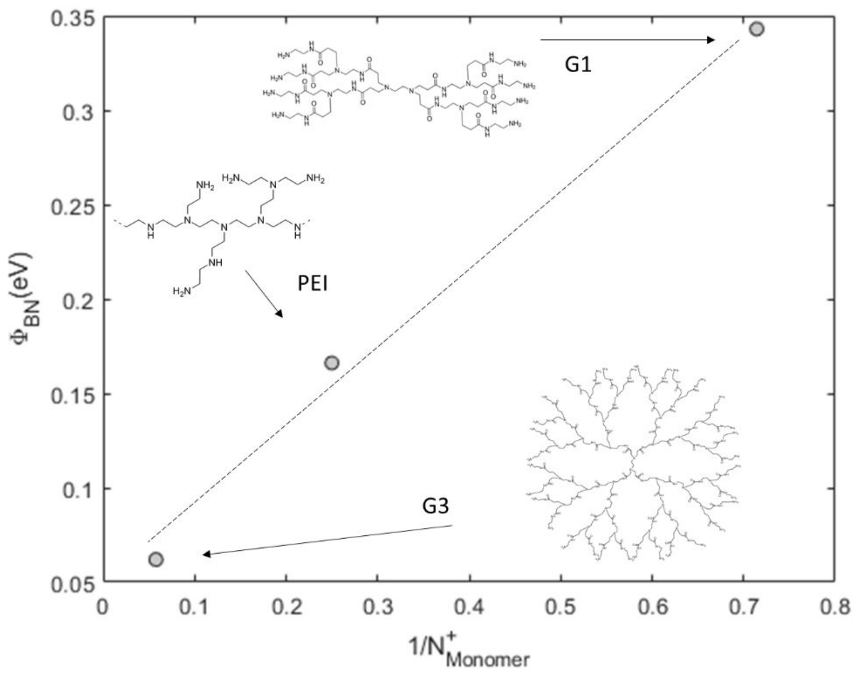

S4- Energy barriers as a function of the inverse of the protonated amino surface groups in each polymer.

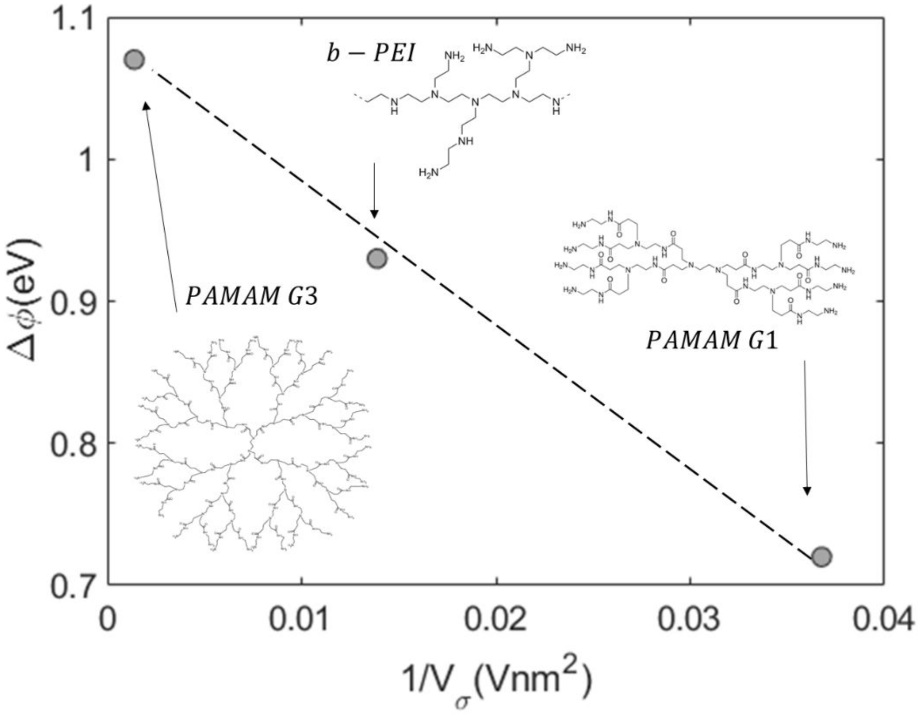

S5- Work function difference with respect to silicon reference as a function of the inverse of the figure of merit.

Structure of cells	*FF* (%)	V_{oc} (mV)	J_{sc} (mA cm^{-2})	*PCE* (%)
ITO/V_2O_5/c-Si/Al (Reference)	64.4	340	28.3	6.2

T1- Photovoltaic parameters of the reference cell (no dipole)

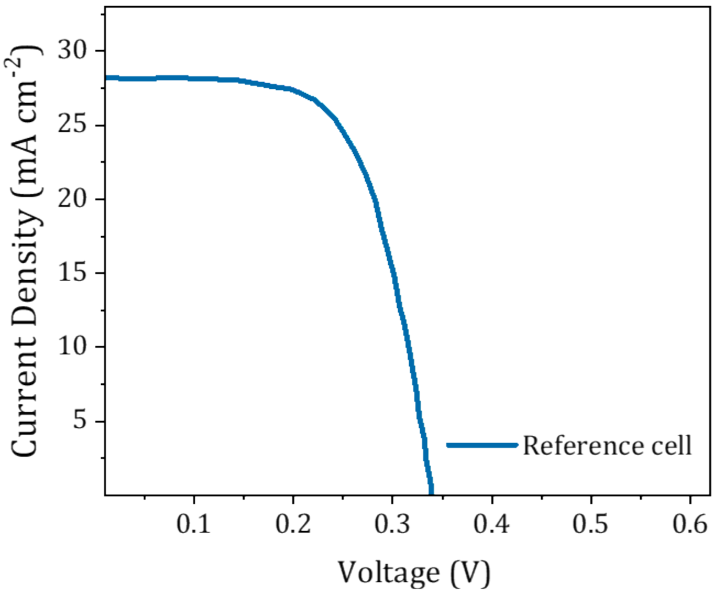

S6- Light IV curve of the reference photovoltaic cell.

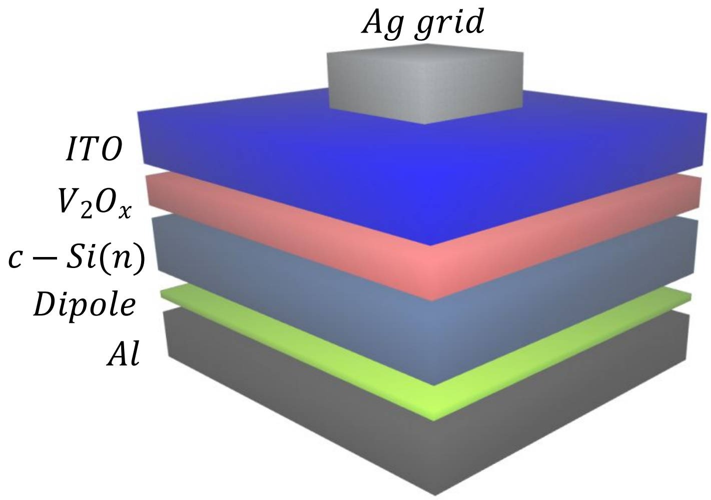

S7-Device structure of the photovoltaic device.
